# C/EBPα Is Dispensable for the Ontogeny of PD-1+ CD4+ Memory T Cells but Restricts Their Expansion in an Age-Dependent Manner

**DOI:** 10.1371/journal.pone.0084728

**Published:** 2014-01-03

**Authors:** Ida Christine Norrie, Ewa Ohlsson, Olaf Nielsen, Marie Sigurd Hasemann, Bo T Porse

**Affiliations:** 1 Finsen Laboratory, Rigshospitalet, Faculty of Health Sciences, University of Copenhagen, Copenhagen, Denmark; 2 Biotech Research and Innovation Center (BRIC), University of Copenhagen, Copenhagen, Denmark; 3 Danish Stem Cell Centre (DanStem) Faculty of Health Sciences, University of Copenhagen, Copenhagen, Denmark; 4 Institute of Biology, University of Copenhagen, Copenhagen, Denmark; Institut National de la Santé et de la Recherche Médicale, France

## Abstract

Ageing and cancer is often associated with altered T cell distributions and this phenomenon has been suggested to be the main driver in the development of immunosenescence. Memory phenotype PD-1+ CD4+ T cells accumulate with age and during leukemic development, and they might account for the attenuated T cell response in elderly or diseased individuals. The transcription factor C/EBPα has been suggested to be responsible for the accumulation as well as for the senescent features of these cells including impaired TCR signaling and decreased proliferation. Thus modulating the activity of C/EBPα could potentially target PD-1+ CD4+ T cells and consequently, impede the development of immunosenescence. To exploit this possibility we tested the importance of C/EBPα for the development of age-dependent PD-1+ CD4+ T cells as well as its role in the accumulation of PD-1+ CD4+ T cells during leukemic progression. In contrast to earlier suggestions, we find that loss of C/EBPα expression in the lymphoid compartment led to an increase of PD-1+ CD4+ T cells specifically in old mice, suggesting that C/EBPα repress the accumulation of these cells in elderly by inhibiting their proliferation. Furthermore, C/EBPα-deficiency in the lymphoid compartment had no effect on leukemic development and did not affect the accumulation of PD-1+ CD4+ T cells. Thus, in addition to contradict earlier suggestions of a role for C/EBPα in immunosenescence, these findings efficiently discard the potential of using C/EBPα as a target for the alleviation of ageing/cancer-associated immunosenescence.

## Introduction

Immunosenescence is a phenomenon commonly observed in elderly people, cancer patients and individuals with chronic infections such as HIV. This condition is due to gradual deterioration of the immune system and causes attenuated response to infections and vaccinations [1], [2], [3]. One of the main contributors to immunosenescence is the functional changes that occur within the T cell compartment, which results in an inefficient immune response. In the immune system of elderly people there is a shift in the CD4+ T cell populations, which leads to fewer naïve T cells and more memory phenotype (MP) T cells and is suggested to be part of the delayed and diminished immune response often found in elderly people [4], [5], [6].

There is compelling evidence that a potent immune response is crucial in protecting and preventing tumor formation [7]. For example, mice deficient in Perforin or INF-γ are more susceptible to tumor formation upon carcinogen exposure, which suggests that an efficient immune response is critical in order to protect against carcinogenesis [8], [9]. A better understanding of the factors involved in the age-dependent and tumor promoting defects in immune cells are important as this may lead to the development of strategies aimed at improving the immune response in the elderly.

The programmed cell death (PD)-1-expressing MP CD4+ T cells have recently drawn some attention, since this population is increasing both during ageing and disease. Furthermore, these cells respond poorly to stimulation [10], [11], [12], [13] and it has therefore been suggested that the attenuated immune response in elderly is a consequence of the accumulation of MP PD-1+ CD4+ T cells. In accordance, blockade of the PD-1 pathway or CTLA-4, another T cell inhibitory molecule, rejuvenates the immune response and improves the overall survival in certain settings [14], [15], [16], [17]. With this in mind, targeting the PD-1+ CD4+ T cell population potentially holds great promise for restoring the immune system in elderly.

Recently, PD-1+ CD4+ T cells were shown to display high expression of the transcription factor CCAAT/enhancer-binding protein alpha (C/EBPα) [12], [18], which is primarily expressed in common myeloid progenitors (CMPs) and required for their differentiation into granulocyte/monocyte progenitors (GMPs) [19], [20]. C/EBPα drives myeloid differentiation by inducing lineage-affiliated gene expression programs and by promoting cell cycle exit [21], [22], [23], [24], 25,26,27. Through these dual activities C/EBPα have the functional properties to act as a master switch between uncommitted proliferating progenitors and cell cycle arrested differentiated cells [28], [29].

In addition to its expression in PD-1+ MP CD4+ T cells, which is suggestive of a function in age/cancer-induced immunosenescence, C/EBPα is also expressed in double negative (DN) 1–4 T cells [18], [30]. However, the overall importance of C/EBPα in T cell development or function has not been addressed previously.

In the present work, we set out to explore the possibility of rejuvenating the immune system by targeting C/EBPα in the PD-1+ CD4+ T cell compartment. In order to do so, we investigated the importance of C/EBPα in lymphopoiesis and in particular in the development of PD-1+ CD4+ T cells. In addition, as the frequencies of PD-1+ CD4+ T cells have previously been suggested to affect the development of leukemia, we tested if leukemic progression was altered in a C/EBPα-deficient context.

## Materials and Methods

### Mice

Animals were maintained at the Department of Experimental Medicine at University of Copenhagen and housed according to institutional guidelines. *Cebpa* ^fl/fl^ and *CD2iCre* mice have been described previously [31], [32]. All experimental animals had been backcrossed for at least 10 generations to the C57BL/6 background.

### Ethics Statement

All animal work was done with approval from the Danish Animal Ethical Committee. This study was approved by the review board at the Faculty of Health Sciences, University of Copenhagen (P12-049).

### Flow Cytometry and Cell Sorting

Thymi from 7–9 weeks old mice were collected and homogenized in PBS +3% FCS. 10×10^6^ cells were incubated with 2 µL Fc receptor block (anti-CD16/32, BD Biosciences) in 100 µL PBS +3% FCS on ice for 5 min, washed in cold PBS +3% FCS and stained with antibodies for flow cytometry. T cell progenitors were stained with antibodies against lineage (Ter119, Mac1, Gr1, B220, CD19, NK1.1, CD3e, CD4, and CD8; e-Bioscience), CD44 (e-Bioscience), and CD25 (BD Biosciences). Mature T cells were stained with CD4, CD3e, and CD8a (e-Bioscience).

BM cells were collected from femur and tibiae by crushing the bones in PBS +3% FCS. Spleens were homogenized in PBS +3% FCS and red blood cells were lysed in BD PharmLyse (BD Biosciences) according to manufactures instructions. B cell progenitors in the BM were stained with antibodies against lineage (Ter119, Gr1, Mac1, CD3e, CD4, NK1.1 (e-Bioscience)), B220 (e-Bioscience), CD43 (BD Biosciences), CD19 (BD Biosciences), IgM (BD Biosciences), AA4.1 (e-Bioscience) and 7-AAD (1 µg/mL, Invitrogen). To detect mature hematopoietic cells, BM and spleen cells were stained with antibodies against Ter119, NK1.1, Mac1, B220, CD8a, CD4, PD-1, CD44, CD62L (e-Bioscience) and DAPI (0,2 µg/mL, Invitrogen). Spleens from leukemic mice were stained with antibodies against CD4 and PD-1 (e-Bioscience), and DAPI (0,2 µg/mL, Invitrogen) was used to discriminate live from dead cells. Samples were run on a LSRII (BD Biosciences) or sorted on a FACSAria (BD Biosciences). Analyses were performed using the software FlowJo (Tree Star Inc.).

### Transplantation Assays

Sublethally irradiated (500 Gy) 12–15 weeks old *Cebpa* ^fl/fl^ and *Cebpa* ^fl/fl^;*CD2iCre* mice were transplanted intravenously through the tail vein with 10.000 GFP positive MLL-ENL primary leukemia cells. Recipient mice were maintained on antibiotics for 2 weeks after transplantation.

### Recombination PCR

To detect the extent of recombination, DNA was purified from relevant cell types and genotyped using the following primers: 5′-CCGCGGCTCCACCTCGTAGAAGTCG-3′, 5′-CCACTCACCGCCTTGGAAAGTCACA-3′ and 5′-GTCCTGCAGCCAGGCAGTGTCC-3′. Band size of 355 bp indicates floxed allele and band size of 560 bp indicates deleted allele.

### qRT-PCR

Total RNA was isolated from PD1+ CD4+ and PD1- CD4+ spleen cells using the RNeasy Mini Kit (Qiagen) and cDNA was generated using the Superscript III Kit (Invitrogen). Gene expression was quantified with real-time quantitative PCR (LightCycler 480, Roche) using Sybr Green (Invitrogen). Expression levels of target genes were normalized to β-actin. Primers used: *Ccnd1* sense 5′-GAACAAGCTCAAGTGGAACC-3′, *Ccnd1* antisense 5′-CTTCAATCTGTTCCTGGCAG-3′, *Cebpa* sense 5′-TGAGAAAAATGAAGGGTGCAG-3′, *Cebpa* antisense 5′-CGGG ATCTCAGCTTCCTGT-3′, *c-Myc* sense 5′-CGAAACTCTGGTGCATAAACT G-3′, *c-Myc* antisense 5′-GAACCGTTCTCCTTAGCTCTCA-3′, *Satb1* sense 5′-ACTGAAACGAG CCGGAATC-3′, *Satb1* antisense 5′-CGGAGGATTTCAGAAAGCAA-3′, *Sostdc1* sense 5′-AACAGCACCCTGAATCAAGC-3′, *Sostdc1* antisense 5′-CAGCCCACTTGAACTCGAC-3′, *Spp1* sense 5′-CCCGGTGAAAGTGACTGATT-3′, *Spp1* antisense 5′-TTCTTCAGAGGACACAGCATTC-3′, *β-actin* sense 5′-TCTTCCAGCCTTCCTTCCT-3′ and *β-actin* antisense 5′-TGCTAGGGCTGTGAT CTCCT-3′, *Ifng* sense 5′- ATCTGGAGGAACTGGCAAAA –3′, *Ifng* antisense 5′-TTCAAGACTTCAAAGAGTCTGAGG-3′, *Il2* sense 5′- GCTGTTGATGGACCTACAGGA-3′, *Il2* antisense 5′-TTCAATTCTGTGGCCTGCTT-3′, *Prf1* sense 5′-GCTCCCACTCCAAGGTAGC-3′, *Prf1* antisense 5′-TTTGTACCAGGCGAAAACTGT-3′, *Gzmb* sense 5′- TGCTGCTAAAGCTGAAGAGTAAG-3′, *Gzmb* antisense 5′-CGTGTTTGAGTATTTGCCCATTG-3′.

### T cell Proliferation Assay

Spleen cells were harvested and red blood cells were lysed with PharmLyse (BD Biosciences) according to manufactures protocol. The splenocytes (50*10^6^/mL) were resuspended in RPMI 1640 medium containing 5 µM Carboxy Fluoroscein Sucinimidyl Ester (CFSE; CellTrace, Invitrogen), incubated for 5 min at room temperature, and then washed 3 times with RPMI1640+10% FCS. Next, the splenocytes were washed in PBS, resuspended in 0,5 mM EDTA and incubated for 5 min at room temperature. Cells were then washed in PBS and resuspended in RPMI 1640 medium supplemented with 10% FCS and 2 µg/mL anti-CD28 antibody (Clone 37.51; e-Bioscience). Subsequently, 1–2×10^5^ cells were seeded in round-bottomed 96-well plates, which had been coated with 1 µg/mL anti-CD3e antibody (Clone 145-2C11; e-Bioscience) for 2 hours at 37°C and washed with PBS. Following 72-hours incubation at 37°C, the cells were washed in PBS, incubated in 0,5 mM EDTA for 5 min at room temperature to remove aggregates, and washed again in PBS. The splenocytes were then stained with antibodies against CD4, washed, and resuspended in PBS +3% FCS prior to flow cytometry analysis on a FACSCalibur (BD Biosciences). Analysis was performed using the software FlowJo (Tree Star Inc.).

### In vivo BrdU Incorporation Assay

Mice were injected with 2 mg BrdU (BD Biosciences) and three hours later the spleens were harvested. Splenocytes were stained with antibodies against CD4 (BD Biosciences) and BrdU according to manufactures protocol (BD Biosciences), run on a LSRII and analyzed using the FlowJo software (Tree Star Inc.).

## Results

### Age- and Leukemia-dependent Increase of C/EBPα Expressing PD-1+ CD4+ T cells

**Figure 1:**
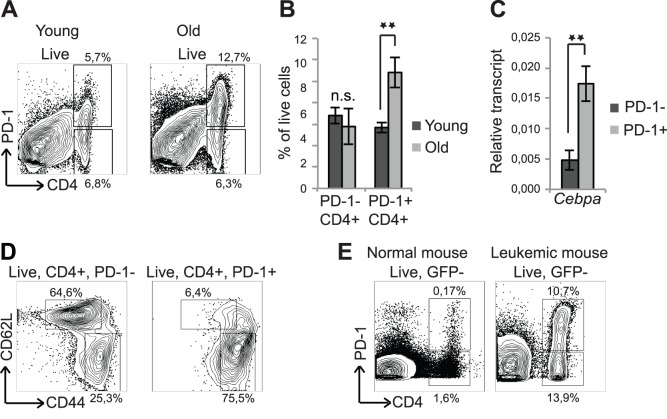
Increase in PD-1+ CD4+ T cells during ageing and in development of AML. (A) Spleen cells from 2 months old and 14 months old mice were stained with antibodies against CD4 and PD-1. (B) Quantification of the data in (A) is presented as mean +/− SD, (young: n = 3, old: n = 7). (C) PD-1- CD4+ and PD-1+ CD4+ splenic T cells from 14 months old mice were analyzed for expression of *Cebpa* normalized to *β-actin* by qRT-PCR. Data are presented as mean +/− SEM, (n = 7). (D) Spleens from 3 months old mice were stained for CD4, PD-1, CD44 and CD62L. A representative example is shown (n = 5). (E) The spleens from healthy (age-matched, non-transplanted) and leukemic mice were analyzed for PD-1+ CD4+ T cells. **P<0.01; n.s.: not significant.

To test the involvement of PD-1+ CD4+ T cells in the depression of the T cell immune response, we first investigated the occurrence of the PD-1+ CD4+ T cells during ageing and leukemia in mice. We therefore harvested spleens from 2- and 14 months old C57BL/6 mice and found the frequencies of PD-1+ CD4 T cells to be increased by 2-fold when comparing old (14 months) with young (2 months) animals, whereas the frequencies of PD-1- CD4+ T cells remained constant (Figure 1A,B). Furthermore, we found the *Cepba* transcript to be prominently upregulated in PD-1+ CD4 *vs.* PD-1- CD4+ T cells (Figure 1C). The PD-1+CD4+ T cell population was mainly restricted to the CD4+, CD44^high^, CD62L^low^ MP population, whereas the PD-1- CD4+ T cells predominantly were CD44^low^, CD62L^high^ (Figure 1D).

Mice with BCR/ABL driven chronic myeloid leukemia display an increase in PD-1+ CD4+ T cells [18] and to test whether this observation could be expanded to other myeloid malignancies such as acute myeloid leukemia (AML) we transplanted bone marrow (BM) cells from an MLL-ENL driven AML mouse into sublethally irradiated recipients. Analysis of the T cell compartment of these animals showed that, similar to CML, AML led to an increase of PD-1+ CD4+ T cells in the spleen (Figure 1E).

Collectively, these findings support previous observations [18] by demonstrating that the accumulation of C/EBPα-expressing PD-1+ CD4+ T cells is a general phenomenon in ageing as well as in leukemia, and therefore implicate C/EBPα as a potential driver of this process.

### C/EBPα is not Important for Maturation of T cells in Young Mice

Whereas C/EBPα is known to play an essential role in the myeloid compartment, its function in the lymphoid lineage has not been investigated in great detail presumably due to its low expression in these cells (Figure S1A) [33], [34]. We therefore generated *Cebpa* ^fl/fl^;*CD2iCre* mice in which C/EBPα is selectively ablated in B- and T cells starting from the common lymphoid progenitor [31], [32] (Figure S1B). The lymphoid compartment of these animals developed normally as assessed by body weight, spleen weight and the cellularities of hematopoietic organs in both young and old mice (Figure S1C,D), suggesting that C/EBPα is dispensable for lymphopoiesis.

**Figure 2:**
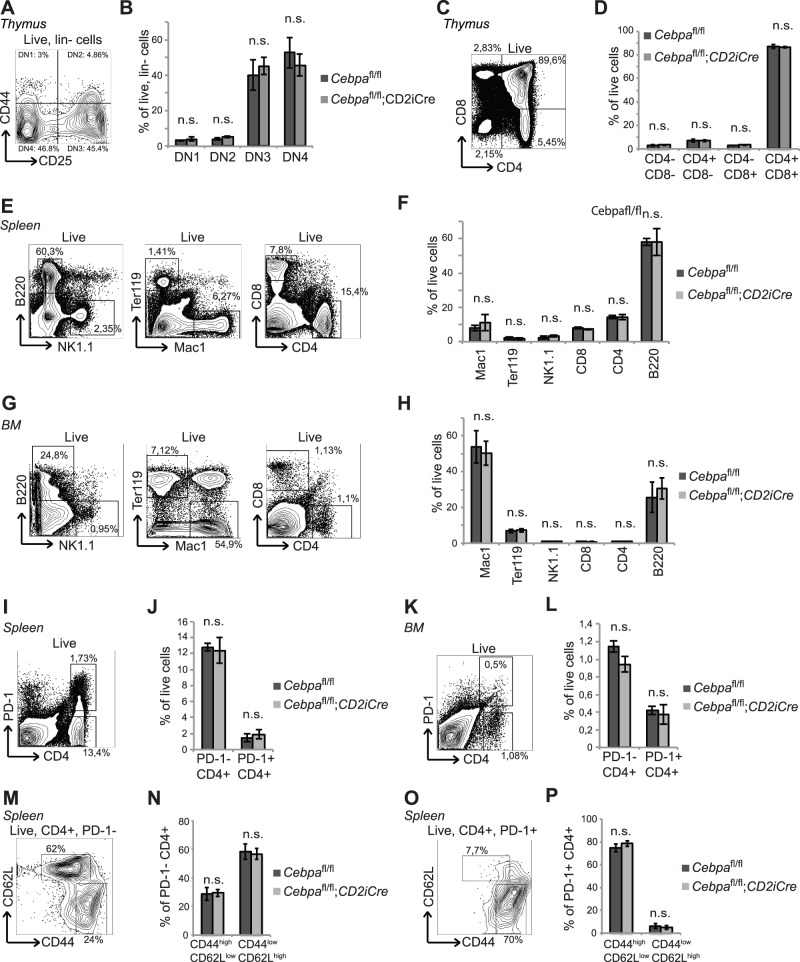
C/EBPα is dispensable for the differentiation of lymphoid cells in young mice. (A and B) Analysis of DN1-DN4 T cells and (C and D) CD4+ and/or CD8+ T cells in thymi from 2 months old *Cebpa* ^fl/fl^ (n = 4) and *Cebpa* ^fl/fl^;*CD2iCre* (n = 5) mice. (E and F) Analysis of mature hematopoietic lineages in spleens from 2 months old *Cebpa* ^fl/fl^ (n = 3) and *Cebpa* ^fl/fl^;*CD2iCre* (n = 4) mice. (G and H) Analysis of the mature hematopoietic lineages in BMs from 2 months old *Cebpa* ^fl/fl^ (n = 5) and *Cebpa* ^fl/fl^;*CD2iCre* (n = 6) mice. (I and J) Analysis of the PD-1+ CD4+ T cells in spleens from 2 months old *Cebpa* ^fl/fl^ (n = 3) and *Cebpa* ^fl/fl^;*CD2iCre* (n = 4) mice. (K and L) Analysis of the PD-1+ CD4+ T cells in BMs from 2 months old *Cebpa* ^fl/fl^ (n = 5) and *Cebpa* ^fl/fl^;*CD2iCre* (n = 6) mice. (M–O) Analysis of CD44 and CD62L subsets within PD-1- and PD1+ CD4+ T cells in spleens from 3 months old *Cebpa* ^fl/fl^ (n = 5) and *Cebpa* ^fl/fl^;*CD2iCre* (n = 5) mice. The contour plots are examples from *Cebpa* ^fl/fl^ mice. Mean +/− SD; n.s. = not significant.

To test if loss of C/EBPα impacted on the early events of T cell development, we isolated thymi from 2 months old *Cebpa* ^fl/fl^ and *Cebpa* ^fl/fl^;*CD2iCre* mice and analyzed the distribution of thymic T cell subsets. We were unable to detect any alternations in the frequencies of DN1-4 cells, CD3+ as well as single and double positive CD4+/CD8+ T cells (Figure 2A–D and Figure S1E), indicating that C/EBPα is dispensable for early T cell development.

Since loss of C/EBPα had no effect on the differentiation of early T cell progenitors, we next examined if C/EBPα-deficiency would affect the differentiation or proliferation of mature B- or T cells in spleen or BM. However, no differences in the expansion of CD4+ and CD8+ T cells, B220+ B cells or NK1.1+ natural killer (NK) cells as well as Ter119+ erythroid cells, or Mac1+ granulocytic/monocytic cells were observed in *Cebpa* ^fl/fl^;*CD2iCre* mice (Figure 2E–H). In addition, no differences in the distribution of pre-pro B cells, pro B cells, pre B cell and mature B cells were observed when comparing BM from *Cebpa* ^fl/fl^ and *Cebpa* ^fl/fl^;*CD2iCre* mice (Figure S2A,B).

Finally, we wanted to investigate whether C/EBPα was responsible for the formation of PD-1+ CD4+ T cells as suggested by Shimatani et al., and therefore analyzed if loss of C/EBPα affected the ontogeny of PD-1+ CD4+ T cells. Surprisingly, we did not detect any changes in the amount of PD-1+ CD4+ T cells (Figure 2I–L), showing that C/EBPα is not required for the formation of PD-1+ CD4+ T cells in neither spleen nor BM of young mice. Furthermore, the frequencies of CD44^high^/CD62L^low^ subsets within the PD-1- and PD-1+ CD4+ T cell compartment were unaffected by deletion of *Cebpa* (Figure 2M–P).

Taken together these results demonstrate that loss of C/EBPα in the lymphoid compartment does not affect the differentiation or distribution of B- and T cells in the thymus, BM, or spleen of young (2 months old) mice.

### C/EBPα Inhibits the Accumulation of PD-1+ CD4+ T cells in the Spleen of Old Mice

**Figure 3:**
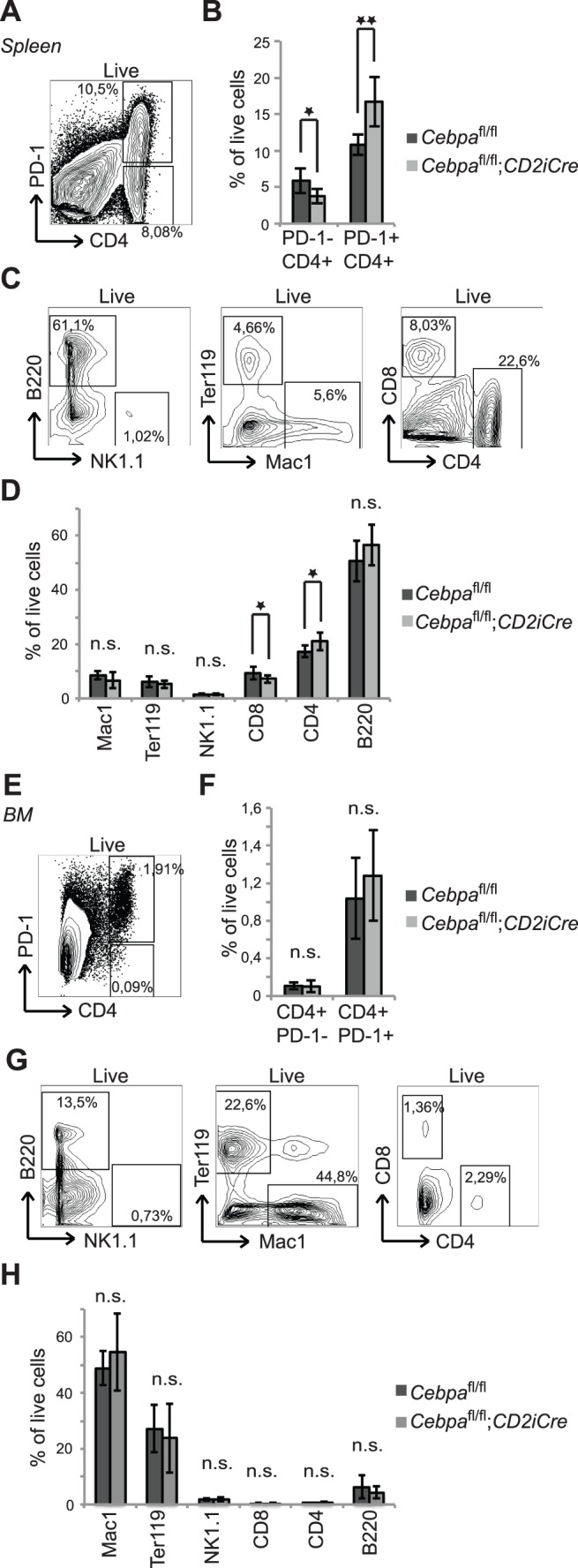
C/EBPα restricts the formation of PD-1+ CD4+ T cells in spleens of old mice. (A and B) Analysis of PD-1+ CD4+ T cells in spleens from 14 months old *Cebpa* ^fl/fl^ (n = 7) and *Cebpa* ^fl/fl^;*CD2iCre* (n = 8) mice. (C and D) Analysis of mature hematopoietic lineages in spleens from 14 months old *Cebpa* ^fl/fl^ (n = 7) and *Cebpa* ^fl/fl^;*CD2iCre* (n = 8) mice. (E and F) Analysis of the PD-1+ CD4+ T cells as well as the mature hematopoietic lineages (G and H) in BMs from 14 months old *Cebpa* ^fl/fl^ (n = 7) and *Cebpa* ^fl/fl^;*CD2iCre* (n = 8) mice. The contour plots are examples from *Cebpa* ^fl/fl^ mice. Mean +/− SD; *P<0.05; **P<0.01; n.s.: not significant.

Given the age-dependent accumulation of PD-1+ CD4+ T cells (Figure 1B), we next tested the possibility of C/EBPα playing a role in the formation or accumulation of PD-1+ CD4+ T cells in old (14 months) mice. Contrary to the observation in young mice, we detected an expansion of PD-1+ CD4+ T cells and a concomitant reduction of the PD-1- CD4+ T cells in the spleen of *Cebpa* ^fl/fl^;*CD2iCre* mice (Figure 3A,B), suggesting that C/EBPα constrains the accumulation of PD-1+ CD4+ T cells specifically in aged mice.

Since we observed this prominent function of C/EBPα in the PD-1+ CD4+ T cells in aged mice, we next tested if C/EBPα loss may also affect the differentiation of other lymphoid lineages in ageing mice. Interestingly, we find that loss of C/EBPα led to a minor, but significant, increase in CD4+ T cells accompanied by a minor decrease in CD8+ T cells in the spleen of aged mice. In contrast none of the other mature cell populations were affected by loss of C/EBPα (Figure 3C,D). Furthermore, we found the changes in frequencies of PD-1+ CD4+ T cells and the CD4/CD8 ratio to be restricted to the spleen as no differences were observed in BMs of 14 months old *Cebpa* ^fl/fl^;*CD2iCre* mice (Figure 3E–H and Figure S2C).

Together, these results suggest that C/EBPα inhibits the accumulation of PD-1+ CD4+ T cells in an age-dependent manner.

### The *in vivo* Proliferation of Aged CD4+ T cells is Restricted by C/EBPα

**Figure 4:**
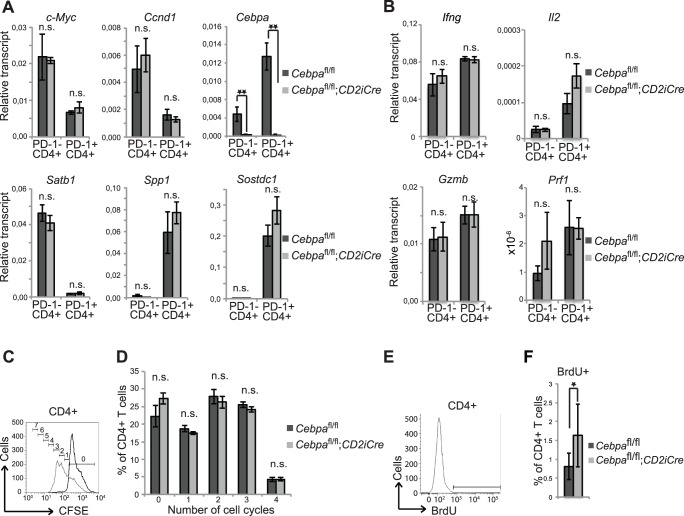
C/EBPα inhibits proliferation of CD4+ T cells in old mice. (A and B) Sorted PD-1- CD4+ and PD-1+ CD4+ T cells from 2 months old *Cebpa* ^fl/fl^ and *Cebpa* ^fl/fl^;*CD2iCre* mice were assessed for transcripts for the indicated genes by qRT-PCR. The relative expression were normalized to β-actin and presented as mean of *Cebpa* ^fl/fl^ n = 7 and *Cebpa* ^fl/fl^;*CD2iCre* n = 8+/− SEM. (C) CFSE labeled splenocytes from 2 months old *Cebpa* ^fl/fl^ and *Cebpa* ^fl/fl^;*CD2iCre* mice were cultured with or without CD3 and CD28 antibodies and after 72 hours after the splenocytes were stained with CD4 antibody and assayed by flow cytometry. Black and grey lines indicate non-stimulated and stimulated cells, respectively. The numbers of cell divisions as given by the Proliferation feature of FlowJo are shown. (D) Quantification of CD4+ T cells in cell cycle 0–4. (*Cebpa* ^fl/fl^ n = 3, *Cebpa* ^fl/fl^;*CD2iCre* n = 3). (E and F) Analysis of proliferation of CD4+ T cells in the spleen of 10 to 15 months old *Cebpa* ^fl/fl^ (n = 8) and *Cebpa* ^fl/fl^;*CD2iCre* (n = 12) mice. The contour plot and histograms are examples from *Cebpa* ^fl/fl^ mice. Mean +/− SD; *P<0.05; **P<0.01; n.s.: not significant.

The work by Shimatani et al. [18] suggested that the senescent features of PD-1+ CD4+ T cells were driven by a C/EBPα-dependent transcriptional program that included the transcriptional inhibition of the proliferation-promoting factors, *c-Myc* and *Ccnd1*, the induction of inflammatory factors such as of *Spp1* and *Sostdc1* and the inhibition of *Satb1*, which represses the expression of PD-1. To test this directly we sorted PD-1+ CD4+ T cells and PD-1- CD4+ T cells from 14 months old *Cebpa* ^fl/fl^ and *Cebpa* ^fl/fl^;*CD2iCre* mice and analyzed the expression of these genes by qRT-PCR. In agreement with Shimatani et al. we observed a reduced expression of *Satb1*, *c-Myc* and *Ccnd1* as well as an increased expression of *Spp1* and *Sostdc1* in PD-1+ vs. PD-1- CD4+ T cells, however the expression was not altered when C/EBPα was deleted (Figure 4A). Similarly, the expression of selected cytokines (Interferon-γ, IL-2, Granzyme B and Perforin) in PD-1+ and PD-1- CD4+ T cells were equally unaffected by the presence or absence of C/EBPα (Figure 4B). Hence, these findings clearly demonstrate that C/EBPα is not responsible for the transcriptional changes that distinguish PD-1+ and PD-1- CD4+ T cells.

Given that C/EBPα can inhibit proliferation through several transcription-independent mechanisms [24], [25], [26], [27] and that ectopic expression of C/EBPα in CD4+ T cells lead to a decreased proliferation [18], we reasoned that C/EBPα might be responsible for the reduced ability of the PD-1+ CD4+ T cells to proliferate upon activation. To test this hypothesis, we harvested spleen cells from *Cebpa* ^fl/fl^ and *Cebpa* ^fl/fl^;*CD2iCre* mice, stained them with CFSE and stimulated with anti-CD3 and anti-CD28 antibodies to induce T cell proliferation. After 72-hours, the cells were stained with antibodies against CD4 and analyzed by flow cytometry for proliferating T cells (Figure 4C,D). Surprisingly, the frequencies of proliferating CD4+ T cells were not affected by loss of C/EBPα, showing that the reduced TCR-mediated proliferation of PD-1+ CD4+ T cells compared to PD1- CD4+ T cells reported by Shimatani et al., was not due to the differences in C/EBPα levels.

Finally, we wanted to examine whether C/EBPα had an impact on the proliferation of PD-1+ CD4+ T cells *in vivo*. We therefore measured the extent of BrdU incorporation in aged *Cebpa* ^fl/fl^ and *Cebpa* ^fl/fl^;*CD2iCre* mice and found a slight increase in the frequency of proliferating CD4+ T cells upon *Cebpa* deletion, which suggests that C/EBPα restricts the proliferation of aged CD4+ T cells (Figure 4E,F).

Taken together, these findings suggest that C/EBPα is responsible for the decreased proliferative capacity of PD-1+ CD4+ T cells, but that it does not affect their expression of signature genes or basal cytokines.

### C/EBPα is Dispensable for the Accumulation of Senescent PD-1+ CD4+ T cells during Cancer Progression

One of the hallmarks in the development of leukemia is immunosenescence, which is believed to contribute to the failure of an effective immune response against cancer cells [35], [36], [37]. Since the PD-1+ CD4+ T cell population increases markedly in aged and leukemic animals (Figure 1), it is therefore likely to contribute to the compromised TCR-response in development of cancer and thus to influence the development of leukemia. Because C/EBPα plays a role in the accumulation of PD1+ CD4+ T cells in old mice, we hypothesized that loss of C/EBPα in the lymphoid compartment may affect the leukemia-dependent accumulation of PD-1+ CD4+ T cells and more importantly, the development of leukemia *per se*.

**Figure 5:**
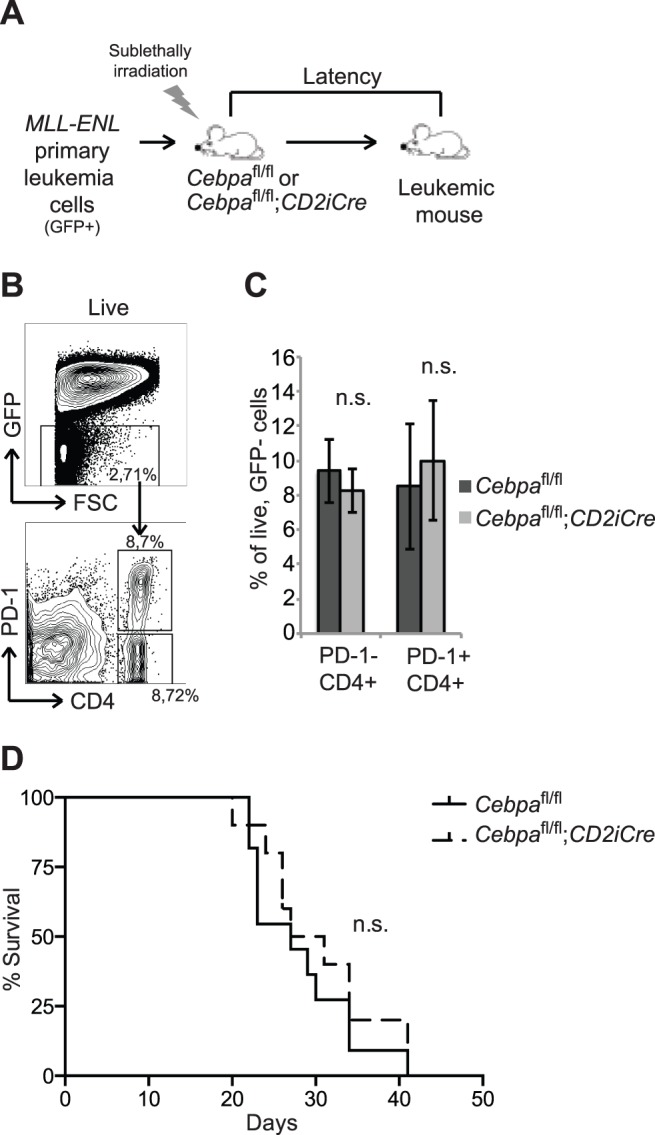
C/EBPα expression in PD-1+ CD4+ T cells does not affect the development of leukemia. (A) Experimental setup. Sublethally irradiated *Cebpa* ^fl/fl^ and *Cebpa* ^fl/fl^;*CD2iCre* recipient mice were transplanted with 10.000 MLL-ENL primary leukemia cells that express GFP. (B) Flow cytometry analysis of GFP- (non-leukemic cells) for the accumulation of PD-1- CD4+ or PD-1+ CD4+ T cells in the spleen as a consequence of leukemic development. (C) Quantification of the data in (B) *(Cebpa* ^fl/fl^ n = 6, *Cebpa* ^fl/fl^;*CD2iCre* n = 9). (D) Survival of the leukemia-transplanted *Cebpa* ^fl/fl^ (n = 11) and *Cebpa* ^fl/fl^;*CD2iCre* (n = 10) mice. The contour plots are examples from *Cebpa* ^fl/fl^ mice. Mean +/− SD. n.s. = not significant.

To test this hypothesis, we first generated primary AML by retrovirus mediated expression of the potent fusion oncogene MLL-ENL, and next transplanted the resulting GFP-positive leukemic cells into sub-lethally irradiated 12–15 weeks old *Cebpa* ^fl/fl^ and *Cebpa* ^fl/fl^;*CD2iCre* secondary recipients (Figure 5A). Following leukemic development, spleens were harvested and the accumulation of PD-1- CD4+ T cells and PD-1+ CD4+ T cells in the recipients GFP negative immune system was analyzed. We detected no differences in the frequencies of PD-1+ CD4+ T cells between the two genotypes, suggesting that loss of C/EBPα has no impact on the accumulation of PD-1+ CD4+ T cells during leukemic development (Figure 5B,C). Moreover, loss of C/EBPα in the lymphoid compartment did not affect disease latency (Figure 5D). Collectively, these findings suggest that C/EBPα is dispensable for the accumulation of PD-1+ CD4+ T cells during disease development and that its loss have no impact on disease progression.

## Discussion

C/EBPα is generally perceived as a myeloid-specific transcription factor involved in the regulation of myeloid vs. lymphoid lineage choices. Indeed, overexpression of C/EBPα in DN1-4 T cells or pre-B cells leads to their trans-differentiation into macrophages and C/EBPα-deficient hematopoietic stem cells upregulate lymphoid gene expression programs [30], [38], [39], [40]. Moreover, a subclass of leukemia patients with silenced C/EBPα expression develops AML with distinct T cell characteristics [41]. Although these findings are consistent with a requirement for the downregulation of C/EBPα during lymphoid development, C/EBPα has also been reported to be expressed in DN1-4 T cells as well as in PD-1+ CD4+ T cells suggesting that C/EBPα could have a previously unrecognized role in lymphopoiesis [18], [30].

In this study we therefore analyzed the potential function of C/EBPα in lymphopoiesis with particular emphasis on a role of C/EBPα in PD-1+ CD4+ T cells and in age/cancer-dependent immunosenescence. Whereas we were unable to detect any changes in the differentiation of B- and T cells in young C/EBPα-deficient mice, aged animals accumulated splenic CD4+ T cells accompanied by a corresponding reduction in CD8+ T cells upon deletion of *Cebpa*. Within the CD4+ T cell compartment, we detected a 50% increase of PD-1+ CD4+ MP T cells in aged C/EBPα-deficient mice, which suggests that C/EBPα potentially restricts the accumulation of these cells in elderly. This is most likely not due to its transcriptional activity since the PD-1+ CD4+ T cells in *Cebpa* ^fl/fl^ and *Cebpa* ^fl/fl^;*CD2iCre* mice have a similar unique transcriptional profile, but rather that C/EBPα appears to inhibit the *in vivo* proliferation of splenic CD4+ T cells.

There is substantial evidence that the occurrence of cancer increases with age. This could be attributed to many processes and pathways including a deregulation of the immune system with age. In particular, the T cell compartment is altered during ageing and is associated with the accumulation of PD-1+ CD4+ MP T cells, a cell population with several senescent features including low proliferation and reduced production of T cell lymphokines following TCR stimulation [10], [11], [12], [13]. Given the accumulation of PD-1+ CD4+ T cells in leukemic mice, as well as the finding that the T cell response can be restored using PD-1 blocking antibodies [15], PD-1+ CD4+ T cells have been suggested to be responsible for the increased susceptibility to disease in elderly [10], [17], [18].

Whereas it is well-established that increased expression of inhibitory molecules, such as PD-1 and CTLA-4 are involved in T cell senescence, the underlying transcriptional mechanisms have not been thoroughly investigated. Recent work has demonstrated a role for the transcriptional repressors BLIMP-1 and FOXP3 in the induction of inhibitory molecules during chronic infections [42], [43], [44], [45]. Thus, deletion of either *Blimp-1* or *Foxp3* alleviates senescent features of T cells [44], [46] demonstrating that these transcription factors are key regulators of immunosenescence. Apart from these findings, we lack knowledge regarding the transcriptional control of immunosenescence, which is needed if we are to develop new strategies for the restoration of T cell response in elderly. In this context it was recently suggested that immunosenescence, and in particular the senescent features of PD-1+ CD4+ T cells, could be attributed to the expression of C/EBPα in T cell subsets. In line with this it was hypothesized that C/EBPα was responsible for the formation of immunosenescent T cells [18], which in turn advocated for C/EBPα as a potential target for the reversal of immunosenescence.

Here we tested these hypotheses in a proper *in vivo* setting using genetically modified mice deficient in C/EBPα in the lymphoid compartment. Our data shows that rather than promoting the accumulation of PD-1+ CD4+ T cells in elderly, C/EBPα specifically limits the accumulation of PD-1+ CD4+ T cells by inhibiting their proliferation. Moreover, the status of C/EBPα does not affect the accumulation of PD1+ CD4+ T cells during leukemic progression and it does not affect the susceptibility to cancer. Interestingly, this may suggest that the accumulation of PD-1+CD4+ T cells in ageing and disease occurs through two independent mechanisms.

In addition to the age-related alterations within the CD4 compartment, it has recently been described that regulatory T cells accumulate in aged mice and cancer patients [47], [48], [49]. Regulatory T cells are important for limiting autoimmune responses, but increasing evidence also suggests a role in dampening the immune response against infections and tumour cells. Importantly, this can be overcomed by depleting regulatory T cells [5], [50], [51]. Whether C/EBPα plays a role in inhibiting the proliferation and accumulation of regulatory T cell subsets remains to be established.

In conclusion, we have analyzed the potential role of C/EBPα during lymphoid development and in immunosenescence. Whereas loss of *Cebpa* only had minor phenotypic impact on general lymphoid development, we find that C/EBPα specifically restricts the accumulation of PD-1+CD4+ T cells during ageing by inhibiting their proliferation. These findings contradict earlier suggestions that C/EBPα promotes immunosenescence and efficiently discard the potential of using C/EBPα as a target for the alleviation of ageing/cancer associated immunosenescence.

## Supporting Information

Figure S1 (A) Gene expression of *Cebpa* in the different hematopoietic lineages. Data were obtained from the HemaExplorer (http://servers.binf.ku.dk/hemaexplorer/) [33], [34]. (B) PCR of recombination in the thymus, spleen and sorted cells from *Cebpa* ^fl/fl^ and *Cebpa* ^fl/fl^;*CD2iCre* mice. fl designates floxed allele, Δ designates deleted allele and * designates an unspecific band. (C) Spleen weight and cellularity of BM, thymus and spleen of 2 months old *Cebpa* ^fl/fl^ (n = 4–12) and *Cebpa* ^fl/fl^;*CD2iCre* (n = 4–12) mice (D) Body weight, spleen weight and cellularity of BM and spleen of 14 months old *Cebpa* ^fl/fl^ (n = 7) and *Cebpa* ^fl/fl^;*CD2iCre* (n = 8) mice. (E) Flow cytometry analysis of immature CD3+ T cells in the thymus from 2 months old *Cebpa* ^fl/fl^ (n = 4) and *Cebpa* ^fl/fl^;*CD2iCre* (n = 5) mice. The contour plot is an example from a *Cebpa* ^fl/fl^ mouse. Mean +/− SD. n.s. = not significant.
(EPS)

Figure S2 **Loss of C/EBPα does not affect early B cell differentiation.** (A) Flow cytometry analysis of immature B cells in BMs of 2 months old or 14 months old *Cebpa* ^fl/fl^ and *Cebpa* ^fl/fl^;*CD2iCre* mice. (B) Quantification of the data in (A) of 2 months old mice (*Cebpa* ^fl/fl^ n = 5, *Cebpa* ^fl/fl^;*CD2iCre* n = 6). (C) Quantification of the data in (A) of 14 months old mice. (*Cebpa* ^fl/fl^ n = 5, *Cebpa* ^fl/fl^;*CD2iCre* n = 6). The contour plots are examples from a *Cebpa* ^fl/fl^ mouse. Mean +/− SD. n.s. = not significant.
(EPS)
